# Epstein-Barr virus infection: the leading cause of multiple sclerosis

**DOI:** 10.1038/s41392-022-01100-0

**Published:** 2022-07-16

**Authors:** Ruirui He, Yanyun Du, Chenhui Wang

**Affiliations:** 1 The Key Laboratory for Human Disease Gene Study of Sichuan Province and the Department of Laboratory Medicine, Sichuan Provincial People’s Hospital, University of Electronic Science and Technology of China, 611731 Chengdu, China; 2 Research Unit for Blindness Prevention of the Chinese Academy of Medical Sciences (2019RU026), Sichuan Academy of Medical Sciences and Sichuan Provincial People’s Hospital, Chengdu, Sichuan China

**Keywords:** Immunological disorders, Neurological disorders

In a recent study published in *Science*, Bjornevik and colleagues demonstrated Epstein-Barr virus (EBV) infection is a trigger for multiple sclerosis (MS) in a longitudinal analysis of more than 10 million US military individuals who were on active duty.^1^

MS is an autoimmune disease that originates in the central nervous system characterized by inflammatory demyelinating lesions. There is no consensus on the etiology of MS. However, we all know that MS is a multifactorial disease which can be influenced by genetic and environmental factors. Genetic factors associated with MS risk are mainly major histocompatibility class II (MHC II) alleles (e.g., HLA-DRB1*15:01, the earliest identified and most dominant risk factor in MS) and MHC I alleles (e.g., HLA-A*02 and HLA B*44, decreasing MS susceptibility). Additionally, more than 200 genetic loci have been identified to be associated with the risk of MS,^2^ but only a few specific genes or biological mechanisms of the identified genetic loci have been studied. Specific environmental exposures including ultraviolet radiation/vitamin D deficiency, cigarette smoking, diet, and viral infections have influence on the incidence of MS. Viral infection is one of the crucial environmental factors and most researches have focused on EBV, a highly B cell-tropic human herpesvirus, which infects about 95% of individuals worldwide. Significantly elevated risk of MS after EBV infection compared with EBV seronegative individuals supports a link between EBV infection and MS.^3^ In addition, anti-EBNAs antibody elevated in blood of MS patients, which can be detected years before MS onset.^4^ Although the hypothesis that EBV causes MS has been studied for many years, clear causality remains inconclusive.

**Fig. 1:**
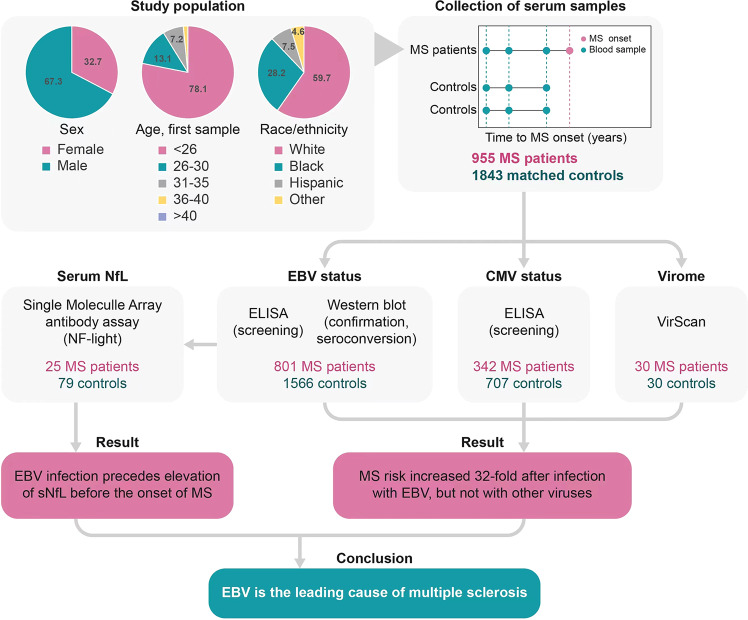
Schematic illustration of the study. Samples from 955 MS case and 1843 controls were randomly selected in the following experiments. Individuals were assessed for EBV and CMV status by three serum samples per person. Serum sNfL levels were measured before, around, and after the time of EBV infection in individuals whose first serum sample was EBV-negative. Virome analysis of serum samples from 30 MS cases and 30 match was made by VirScan. These experiments suggest that EBV is the leading cause of multiple sclerosis

In this study, Bjornevik et al. gave direct evidence that EBV infection is a critical contributor of MS based on a large cohort of more than 10 million young adults in the US military. Among this large population, 955 individuals of them were diagnosed with MS during their active-duty. In a 20-year collaboration with US military between 1993 and 2013, the authors collected up to three serum samples for each MS case before the date of disease onset (Fig. 1). At the same time, each MS case was matched to two individuals without MS who were randomly selected but with the same sex, age, race/ethnicity, kinds of military service, and the collection dates of blood samples (Fig. 1). Removing serum samples insufficient for EBV serology, they got a reasonable sum of samples from 801 cases of MS and 1566 matched controls that could be used to assess EBV infection status and 35 MS cases were identified as EBV-negative at the baseline (Fig. 1).^1^ In order to clarify the relation of EBV infection and MS onset, EBV status of the 35 MS cases and 107 corresponding controls were analyzed during the follow-up. A significantly higher seroconversion (97% rate contrasts with the 57% rate of seroconversion in the third serum samples) was found in the individuals who developed MS.^1^ However, seroconversion rates for cytomegalovirus (CMV), the negative control virus, were comparative between the population who had MS symptoms later and others who did not have.^1^ Moreover, by comparing EBV seroconversion and EBV-positive rates versus EBV-negative rates, the authors found that risk ratio for MS were 32.4 and 26.5, respectively.^1^ Collectively, these data confirmed that EBV infection happens before MS onset and further revealed the significant association of EBV infection and higher risk of MS.

Serum neurofilament light chain (sNfL) is a sensitive biomarker of neurodegeneration, and may be also a biomarker of initiation time of MS process . To further clarify the temporal correlation between EBV infection and MS, Bjornevik et al. detected concentrations of sNfL at different time points of EBV infection in serum samples from 25 MS cases and 79 controls (Fig. 1).^1^ They found that sNfL levels significantly increased in MS cases than in controls only after EBV infection compared with before EBV infection. Thus, the results further indicated that EBV infection happens earlier than sNfL elevation before MS onset.

There is also a question that whether immune disorder in the preclinical phase of MS were able to increase susceptibility more generally to virus infection and further aggravate the disease. The authors randomly selected 30 cases of MS and 30 matched controls with two serum samples, collecting before and after the onset of symptoms (Fig. 1).^1^ Moreover, the VirScan data of serum samples showed similar antibody response of MS patients and controls to viral peptides at two time points, except for EBV.^1^ This finding thus argues against the possibility that a second hit from other viruses may have important influence on MS etiology and further supports that EBV infection is specifically associated with MS risk.

MS includes three clinical stages: a pre-clinical stage detected only by MRI; a relapsing-remitting (RR) stage characterized by a marked relapse and remission course; and a progressive stage usually evolving from the relapsing stage. EBV likely involves in the entire clinical course of MS, and may play different roles in MS different stages. With the understanding of the immune mechanism of MS, more than ten immunotherapy drugs for MS have been approved by FDA. Anti-CD20 monoclonal antibody has become one of the most effective treatments for MS.^5^ However, anti-CD20 monoclonal antibody does not exhaust the progeny of its targeting circulating memory B cells and plasma cells which lack CD20 expression. Moreover, Due to the blood brain barrier (BBB), it is difficult for anti-CD20 antibody to freely reach the central nervous system. Therefore, antiviral therapy directly targets EBV may be a new opportunity for MS therapy. Several anti-herpes virus compounds, including famciclovir and abacavir, have been evaluated for the treatment of MS, but they have shown poor therapeutic efficacy for MS. It is worth noting that FDA has approved human IFN-β for relapsing MS treatment. The mechanism by which IFN-β treats MS is unclear, but it is known that IFN-β can inhibit EBV infection. In addition, EBV negative individuals have very low risk of MS suggests that MS risk is associated with EBV infection and a vaccine against EBV could be developed to prevent MS in future.

Overall, this study suggests that the occurrence of EBV infection is a cause but not a consequence of MS. Moreover, these results cannot be explained by any known risk factor for MS and demonstrate that EBV is the leading cause of MS.
